# Design and implementation of an illumination system to mimic skyglow at ecosystem level in a large-scale lake enclosure facility

**DOI:** 10.1038/s41598-021-02772-4

**Published:** 2021-12-06

**Authors:** Andreas Jechow, Günther Schreck, Christopher C. M. Kyba, Stella A. Berger, Lukas Thuile Bistarelli, Matthias Bodenlos, Mark O. Gessner, Hans-Peter Grossart, Franziska Kupprat, Jens C. Nejstgaard, Andreas Pansch, Armin Penske, Michael Sachtleben, Tom Shatwell, Gabriel A. Singer, Susanne Stephan, Tim J. W. Walles, Sabine Wollrab, Karolina M. Zielinska-Dabkowska, Franz Hölker

**Affiliations:** 1 Department of Ecohydrology, Leibniz Institute of Freshwater Ecology and Inland Fisheries (IGB), Müggelseedamm 310, 12587 Berlin, Germany; 2 Department of Experimental Limnology, Leibniz Institute of Freshwater Ecology and Inland Fisheries (IGB), Alte Fischerhütte 2, 16775 Stechlin, Germany; 3 Remote Sensing and Geoinformatics Section, Helmholtz Center Potsdam, German Center for Geosciences (GFZ), Telegraphenberg, 14473 Potsdam, Germany; 4 Berlin-Brandenburg Institute of Advanced Biodiversity Research (BBIB), Königin-Luise-Str. 2-4, 14195 Berlin, Germany; 5 Department of Ecology, Berlin Institute of Technology (TU Berlin), Ernst-Reuter-Platz 1, 10623 Berlin, Germany; 6 Institute for Biochemistry and Biology, University of Potsdam, Maulbeerallee 2, 14469 Potsdam, Germany; 7 Department of Lake Research, Helmholtz Centre for Environmental Research (UFZ), Brückstr. 3a, 39114 Magdeburg, Germany; 8 Department of Ecology, University of Innsbruck, Technikerstrasse 25, 6020 Innsbruck, Austria; 9 GUT Light Lab, Faculty of Architecture, Gdańsk University of Technology (Gdańsk Tech), Narutowicza 11/12, 80-233 Gdansk, Poland; 10 Institute of Biology, Freie Universität Berlin, Berlin, Germany

**Keywords:** Optics and photonics, Limnology

## Abstract

Light pollution is an environmental stressor of global extent that is growing exponentially in area and intensity. Artificial skyglow, a form of light pollution with large range, is hypothesized to have environmental impact at ecosystem level. However, testing the impact of skyglow at large scales and in a controlled fashion under in situ conditions has remained elusive so far. Here we present the first experimental setup to mimic skyglow at ecosystem level outdoors in an aquatic environment. Spatially diffuse and homogeneous surface illumination that is adjustable between 0.01 and 10 lx, resembling rural to urban skyglow levels, was achieved with white light-emitting diodes at a large-scale lake enclosure facility. The illumination system was enabled by optical modeling with Monte-Carlo raytracing and validated by measurements. Our method can be adapted to other outdoor and indoor skyglow experiments, urgently needed to understand the impact of skyglow on ecosystems.

## Introduction

Emissions of artificial light at night (ALAN) have dramatically increased since the early twentieth century, and current growth rates of lit area and light intensities on Earth exceed 2% per year at the global scale^1^. ALAN makes it possible to extend human activities (both indoor and outdoor) into the night, and is asociated with wealth and safety, rendering public perception of ALAN overwhelmingly positive^2^. However, long aware of the artificially increasing brightness of night skies, astronomers have recognized negative aspects of ALAN, pointing to the ‘dark side’ of light emissions by coining the term light pollution^3^. Impacts of ALAN on life were also recognized early on^4^, but detailed investigation of the impact of ALAN on life only began during the last two decades^5–8^ showing effects on species level ranging from microorganisms^9^ to mammals^10^ as well as on important ecosystem services such as pollination^11^. Because humans tend to settle near water bodies^12^, aquatic environments are particularly affected by ALAN, but are currently understudied compared to terrestrial ones^9,13–15^.

**Figure 1:**
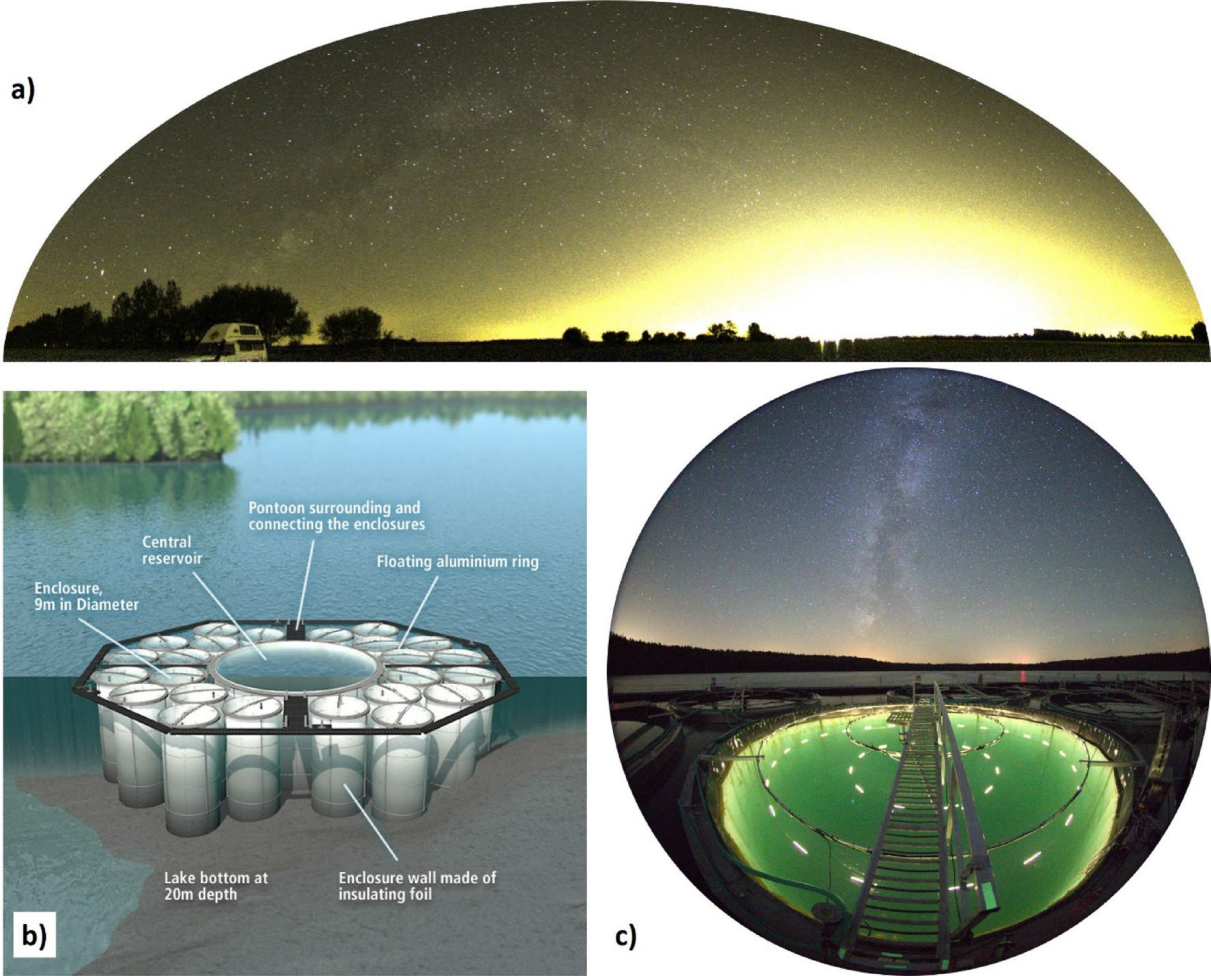
The skyglow illumination system. (**a**) Panoramic image of the skyglow above the city of Berlin, Germany, viewed from 30 km distance obtained from an all-sky image. This image was not taken at the experimental site and serves for illustration purpose of the phenomenon of skyglow. (image credit: A. Jechow) (**b**) Sketch of the enclosure facility, LakeLab, in Lake Stechlin, Germany (image credit IGB, www.lake-lab.de). (**c**) Skyglow system illuminating one of the enclosures of Lake Lab. The Milky Way and weak skyglow from small towns at the horizon are visible in the background. The green appearance of the water results from backscattering of the LED light. (image credit: A. Jechow).

Artificial skyglow^16^ is a form of light pollution which is caused by ALAN being scattered within the atmosphere and redirected back towards the Earth surface^17^. It causes the diffuse glow above illuminated areas such as a large city (Fig. 1a). Skyglow extends over large areas^18^ and can be sensed over large distances ranging from several tens to hundreds of km away from the light sources^19,20^. In urban areas, skyglow for clear sky condition can exceed the natural night sky brightness by factors of several tens to hundreds^20–23^ resulting in ground illuminances (in the horizontal plane) on the order of 0.01–0.1 lx. Skyglow is dramatically amplified during overcast conditions^20,24^ and can then reach extreme night sky brightness values and illuminances of more than 1 lx^20–23^, which is much brighter than moonlight^25^.

Although it is suspected that ecological impacts of skyglow are significant^5–7,26^, experimental studies are scarce. For example, the remarkable sensitivity of the circadian system of several taxa in lab studies, including common freshwater fish^27,28^, indicate that skyglow could influence wild animals over large areas. Another recent study suggests that low skyglow-like ALAN levels disrupt the migration of Talitrus saltator (commonly known as sandhoppers), that normally use celestial light at night for orientation^29^. Finally, the amplitude of diel vertical migration of zooplankton can be affected by skyglow, as shown in a seminal experiment conducted in small enclosures deployed in an urban lake^30^.

However, an experimental demonstration of the effects of skyglow in a controlled fashion at a larger scale or at ecosystem level remained elusive so far. Demonstrating ecosystem impacts of environmental stressors requires experiments in realistic settings and of sufficient duration, and the assessment of light pollution effects is no exception. Consequently, experimental test sites have been established to assess consequences of direct ALAN exposures, mainly street lighting^31–33^. Experiments on skyglow, however, are challenging because the spatial light field of the diffuse uniform illumination of skyglow is difficult to emulate, lighting structures shade natural light, and dark experimental control units can be accidentally illuminated^34^. One possible approach is to document ecological responses before and after switching off skyglow sources at the whole-village or city scale. However, such experiments are difficult to implement and virtually impossible to replicate, notably in view of the highly dynamic nature of artificial skyglow resulting from variation in cloud cover^35^, ground albedo^23^ or changes in urban light emissions^35–37^. Moreover, such experiments can test for recovery from skyglow, the control treatment being the illuminated city, but not on skyglow effects on naïve communities and ecosystems, that never experienced ALAN before. Similar limitations apply to experiments taking advantage of world-wide mass events like the WWF Earth Hour^37^. Thus, city-scale experimental approaches can be valuable to study short-term impacts of skyglow (hour scale), or release thereof, for instance on animal behavior, but cannot inform about long-term consequences on ecosystem-level responses.

Here, we present the first large-scale experimental setup that mimics skyglow realistically at ecosystem level for ALAN naïve communities at a lake enclosure facility located in an area with very low skyglow^18^. The skyglow system was modeled with Monte Carlo raytracing including underwater light propagation and then implemented in the field using concentric rings of light emitting diodes (LEDs) with spatially diffuse emission and a warm-white light spectrum. We outline the rationale behind the design of the skyglow setup and provide details of the implementation and performance of the installation.

## Results

A large-scale experimental enclosure facility implemented in a freshwater lake (Fig. 1b) was illuminated to mimic skyglow (Fig. 1c) on a freshwater ecosystem utilizing state of the art optical modeling. For more information on the facility, the location and its night sky brightness, the experimental requirements, and practical limitations of the skyglow illumination system see Methods section. Figure 1a illustrates the phenomenon of skyglow and was not obtained at the location of the facility, but also near the city of Berlin.

### Modeling the ring geometry: emitters in radial direction

**Figure 2:**
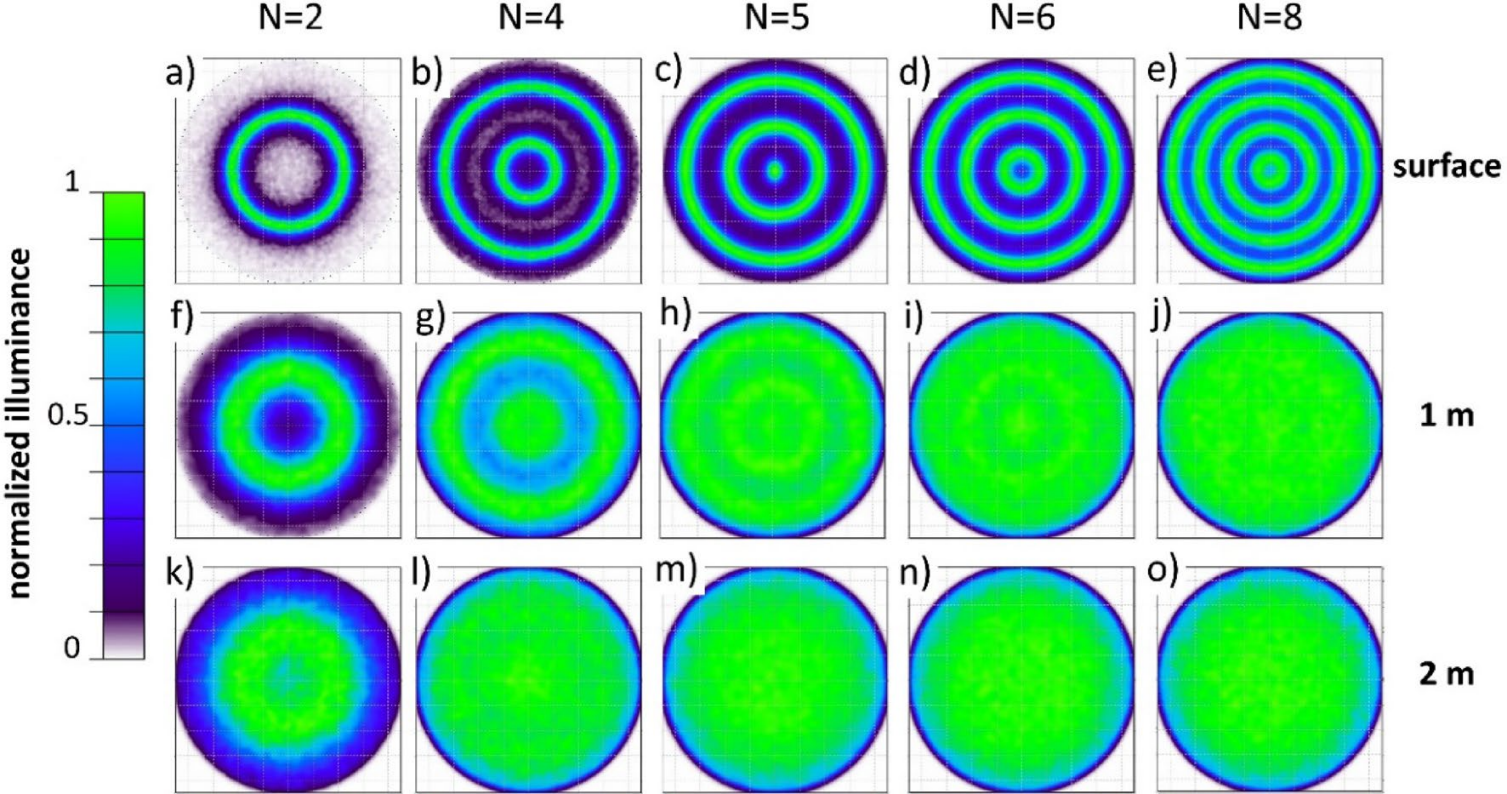
Simulation results top view. Modeled horizontal illuminance distribution of the skyglow system for different water depths and for different geometries of ring-shaped light emitters. The upper row (**a**-**e**) shows the light distribution at the water surface, the middle row (**f**-**j**) the light distribution at 1 m and the lower panels (**k**–**o**) at 2 m below the water surface.

Given the circular symmetry of the enclosures, a circular shape of the illumination system using a ring-shaped construction was considered most effective. However, given the much more simplistic manufacturing of a rectangular structure, the ring geometry was tested against a rectangular geometry and found to be more effective (Figs. S1, S2). Subsequently, different ring geometries were modeled to find out how many emitters N across the enclosure diameter are needed for a homogeneous light distribution. The starting parameters for the optical simulation of this arrangement were defined in terms of size of the rings and the number of light emitters N encountered while crossing the diameter of the enclosure (Fig. S3). In this terminology, N = 1 is a single emitter in the center, N = 2 is two emitters across the diameter or one ring (Fig. 2a), N = 3 is one ring plus a single emitter in the center, N = 4 is two rings (Fig. 2b), N = 5 is two rings plus a central emitter (Fig. 2c) and so on. The horizontal illuminance distribution for five selected ring geometries with different numbers of emitters N are shown in Fig. 2. The upper row (a-e) shows the light distribution at the surface, the middle row (f-j) the light distribution at a depth of 1 m and the lower row (k–o) at 2 m depth. In all these models, the whole surface area of each ring is a light emitter, radiating light towards the water surface with the ring-shaped light emitters positioned at z = 0.4 m above the water surface.

The homogeneity of the light field created by a single ring (i.e. N = 2) was largely insufficient (Fig. 2a,f,k), showing a peak-to-valley ratio (PVR = E_max_/E_min_) of the illuminance at the water surface of PVR > 20, which reduced to PVR ≈ 7 at 1 m depth. The double-ring geometry with N = 4 emitters (Fig. 2b,g,l), greatly improved the peak-to-valley ratio to PVR ≈ 9 at the surface and to PVR ≈ 1.5 at 1 m depth. The addition of a central emitter to the double-ring geometry with N = 5 (Fig. 2c,h,m) provides an improved peak-to-valley ratio of PVR ≈ 6 at the surface and PVR ≈ 1.3 at 1 m depth, which is already relatively homogeneous. Adding one more emitter across the diameter with N = 6 (Fig. 2d,i,n) gives only a minor improvement and the four-ring geometry with N = 8 (Fig. 2e,j,o) results in the lowest PVR of about 2 at the surface but only little further improvement at 1 m depth compared to the double ring plus central emitter version (N = 5). All solutions apart from N = 2 provide a good homogeneous light field at 2 m depth. These results indicate that the solution with N = 5 is the best compromise of lowest material use, blocking of natural light by providing already a good homogeneity at about 1 m depth.

### Influence of wall albedo

**Figure 3:**
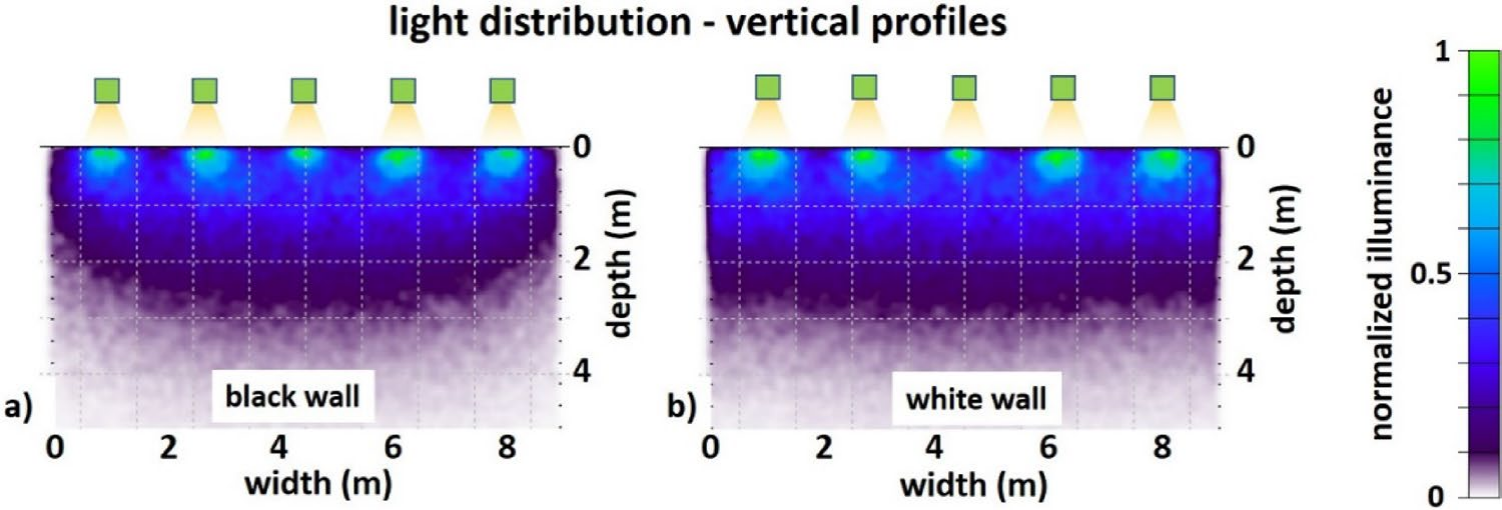
Simulation results side-view. Illuminance distribution in the vertical plane for different wall reflectivities. (**a**) a highly absorbing wall (black), (**b**) a Lambertian wall with high albedo (white).

The spatial light distribution at a given depth does not only depend on scattering and absorption within the bulk water body, but also on losses, reflections and scattering at the enclosure walls. To assess the influence of the wall albedo, we modeled two extreme cases: a black wall that fully absorbs the incident light, and a white wall with 99% Lambertian diffuse reflectance (Fig. 3). Compared to the black absorbing wall (Fig. 3a), the white diffusely reflecting wall (Fig. 3b) improved uniformity of the light field particularly near the edges of the enclosures. This is most apparent at the enclosure walls at a depth lower than 3 m, where the light intensity was lower than in the center of the enclosure when using the black wall (Fig. 3a) but became almost homogeneous in the horizontal plane when using the white wall material (Fig. 3b).

### Emitters in azimuthal direction

Thus far, the light distribution was segmented only in the radial direction, while in the azimuth direction continuous illumination along the ring-shaped emitters was modeled. However, the simulation of the ring geometry (Fig. 2) showed that an emitter spacing of 1.8 m between the rings will give an acceptable light distribution in the radial direction. Thus, a segmentation of the ring-shaped emitters along the azimuth direction is considered as well. Practical boundaries arise from the length of the emitters and the luminous flux of the LED strips which was 130 lm per m length. The preferred setup with N = 5 emits light from two rings with 3.6 m and 7.2 m in diameter, giving a total length of the light-emitting structure of ca. 34 m which emits a total luminous flux of about 4,400 lm. With the surface area of the enclosure of 64 m^2^, this would result in an average illuminance of 69 lx at the water surface, which is larger than the targeted 0.01–10 lx for the skyglow treatment (see Methods section). The LED strips are available at a minimum length of 27.5 cm and a spacing of 94 cm along the azimuth axis corresponds to 12 emitters for the small and 24 for the large ring plus one central emitter for N = 5. This segmentation reduces the total emitter length to ca. 10.2 m, the total luminous flux to 1330 lm and the maximum illuminance at the water surface to 21.1 lx, respectively.

**Figure 4:**
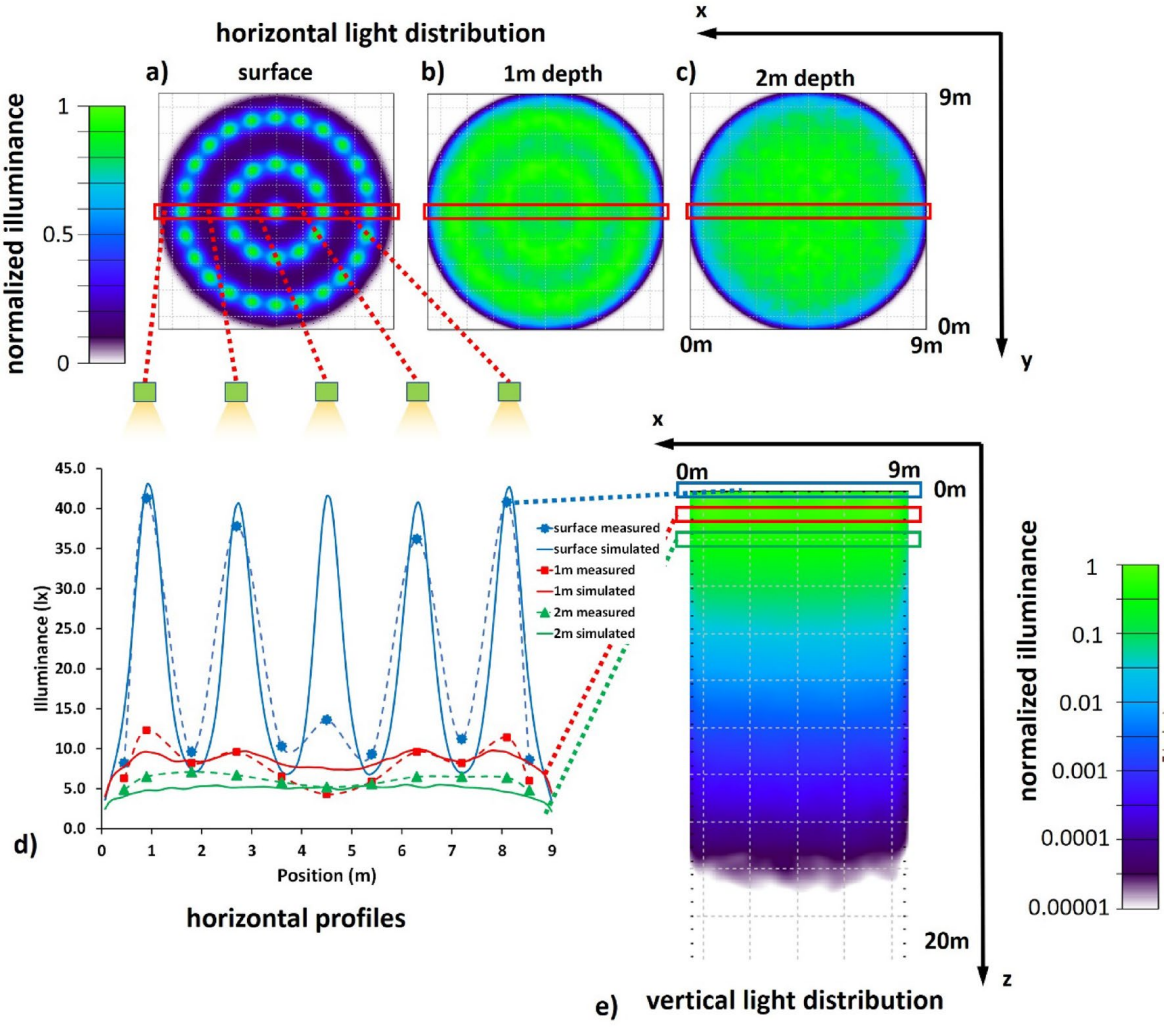
Simulation and measurements final design. Illuminance distribution of the final design of the skyglow illumination system simulated for a segmented ring geometry and a total of 37 LED strips: horizontal light distribution (**a**) at the water surface, (**b**) 1 m and (**c**) 2 m below the water surface. (**d**) horizontal profiles and measured illuminance and (**e**) vertical light distribution in a cross section of the center of the enclosure down to 20 m depth.

The geometry with the 37 emitters was modeled and compared with the original N = 5 full ring geometry (Fig. S4 for direct comparison). Each emitter was implemented in the CAD model and a luminous flux of 36 lm was applied per emitter. The sectioning along the azimuth is clearly perceivable at the water surface (Fig. 4a vs. Figure 2c, also Fig. S4a,b) but the PVR did not increase. At 1 m (Fig. 4b) and 2 m (Fig. 4c) water depth the light distribution with the 37 emitters becomes indistinguishable from the double ring plus central emitter version.

### Testing and optimizing the final setup

Based on these modeling results and practical considerations (see Methods section), we chose the design with N = 5 and the segmented light emission with 37 total emitters and the white surface for the enclosure walls for the final design of the illumination system. To validate the modeling, we measured the horizontal illuminance with a sensitive underwater luxmeter (ILT 1700 with SUD 33 photopic cosine corrected detector, International Light Technologies, Peabody, USA) at different positions across the diameter of the enclosure at the surface and different depths. The measurements were performed during a moonless night to avoid additional natural light disturbing the result. The natural background was determined to be in the range of the detection limit of the instrument of 0.001 lx. Because of geometrical constraints of the enclosure, the emitters were mounted at slightly different heights, with the large ring closer to the water (25 cm) than the small ring (40 cm) and the central emitter at the largest distance (45 cm). This led to an inhomogeneous intensity distribution for this setup operated at equal luminous flux and without additional wall material (Fig. S5). In this configuration, the illuminance was smallest at the center, largest underneath the large ring and the intensity modulation reached high values of PVR ≈ 200 at the water surface. The measured illuminance of 21.1 ± 29.6 lx at the surface was in the range of the 21 lx estimated beforehand. Even this unoptimized setup showed improved homogeneity at the depths of 1 m (PVR ≈ 3) and 2 m (PVR ≈ 2).

Individual dimming adjustments of the LEDs on the large ring and installation of the white wall material improved homogeneity of the light distribution. The measurements and modeling results for this optimized design are plotted in Fig. 4, showing horizontal illuminance distribution at different depths (Fig. 4a–c) and the horizontal illuminance distribution across the enclosure (Fig. 4d) as well as the vertical distribution of the light field (Fig. 4e—note the log scale of the illuminance). The horizontal profiles (Fig. 4d) showed a relatively good match between modeling (solid lines) and measurement results (dashed lines) with a deviation at the central emitter. For this configuration, the illuminance reduced to 16.9 ± 12.9 lx (SD) at the surface. The intensity modulation improved to PVR ≈ 25 at the water surface, to PVR ≈ 2 at 1 m depth and to PVR ≈ 1.2 at 2 m depth.

**Figure 5:**
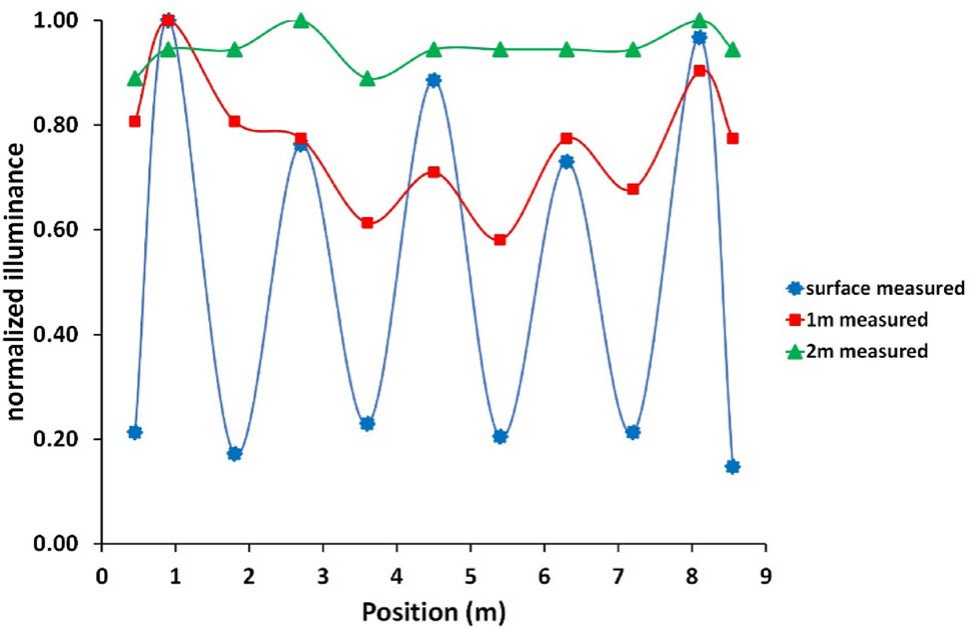
Illuminance measurement of final design. Normalized illuminance distribution measured at the surface and 1 m and 2 m water depth for the 6 lx extreme skyglow treatment with adjusted dimming. For numerical values see table S2.

Further dimming was applied to implement the two skyglow treatments for the experiments with an extreme skyglow treatment at 6 lx and a standard skyglow treatment at 0.06 lx, respectively. Figure 5 shows illuminance measurements obtained with system set to the higher 6 lx skyglow treatment, the results were normalized to 1 for better comparison of homogeneity between different depths. The dimming adjustments resulted particularly in an improved homogeneity at the surface with a measured illuminance of 6.2 lx ± 4.1 lx and an improved homogeneity of PVR ≈ 6. At 1 m depth, the homogeneity improved slightly to PVR ≈ 1.7 and a near homogeneous light distribution with a PVR ≈ 1.1 was achieved at 2 m depth. For the lower skyglow treatment, the system was further dimmed by a factor of 100 (see dimming curve in Fig. S6) and validating by light measurements at a depth of 1 m. Sporadic tests of the light distribution were obtained several times during each of the experiments and checked for plausibility by using measured diffuse attenuation coefficients and comparing illuminance at different depths.

### Practical implementation

**Figure 6:**
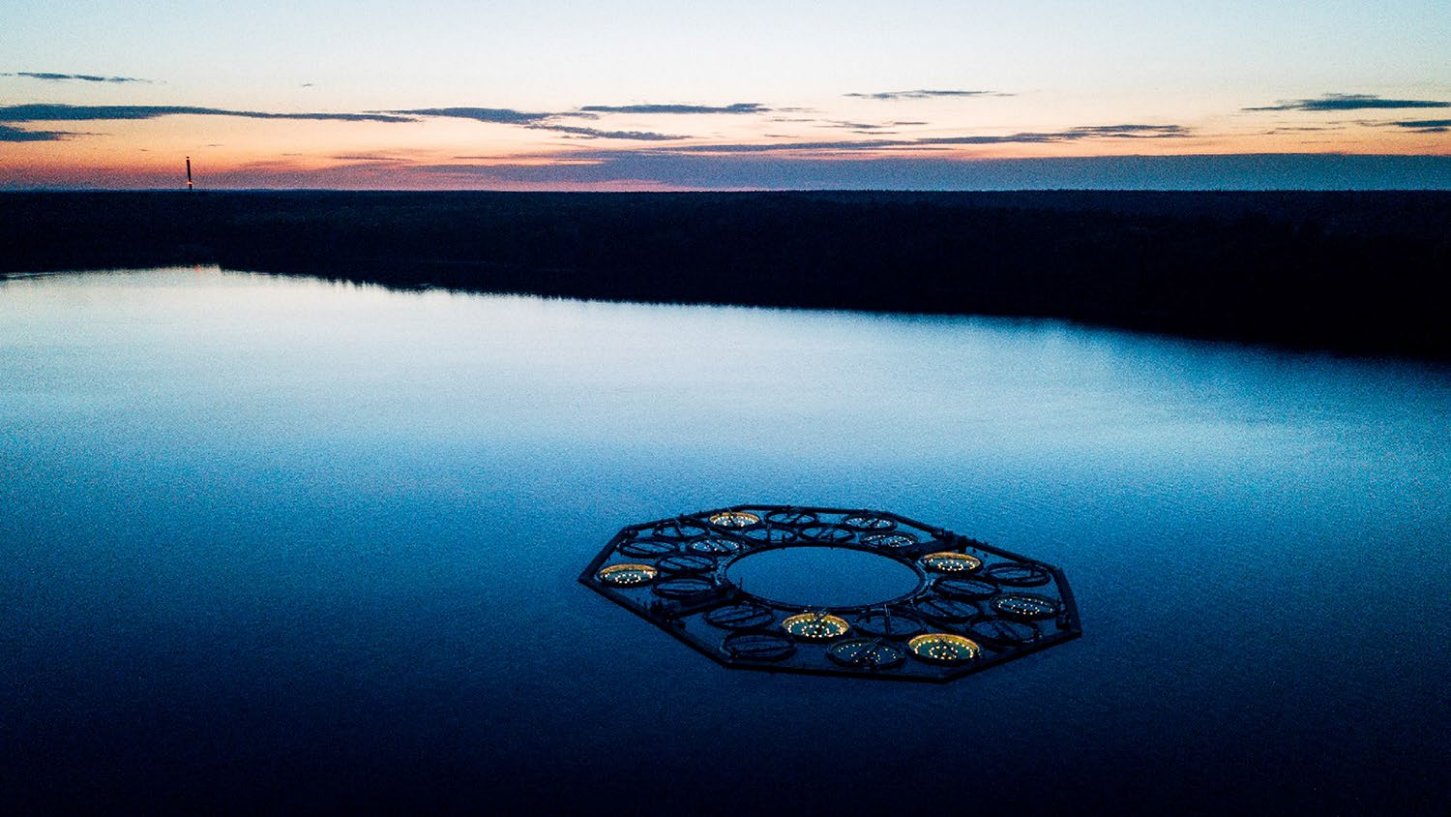
The illuminated LakeLab. Aerial photograph of the LakeLab in Lake Stechlin, Germany, with five enclosures each illuminated by high and low levels of emulated skyglow, respectively. (image credit: V. Crone).

Identical fully functional light sources, consisting of LEDs arranged equidistantly on concentric rings, were installed in all 24 enclosures of the LakeLab (see Fig. 1c for a single enclosure and Fig. 6 for an aerial view of the facility). This ensured perfect flexibility in the allocation of treatments to the experimental units and precluded any unintended systematic influences of the illumination system (e.g. leaching of chemicals from the installation) on the experimental outcomes. Instead of “dark” controls, the controls were exposed to natural light^34^. The final design had a double ring geometry (large and small ring plus single central emitter) with 37 emitters per enclosure (see "Results"). None of the 888 LED strips showed any degradation about 3 years after installation outdoors. Light emitters were mounted on an aluminum construction that consisted of rectangular aluminum tube (40 mm height × 20 mm width, with 2.0 mm wall thickness; Fig. S7a) that was bent to the calculated diameters in segments of about 5.6 m length. The metal pieces were cleaned, rinsed, and kept in lake water for several days before joining them to complete rings. The LED strips were attached on land (Fig. S7b) using UV resistant cable ties and an additional aluminum U-profile for extra mechanical support and to provide additional shielding of residual horizontal light. The rings were then transported to the site with a barge (Fig. S7c). The small ring and the central emitter were mounted beneath the bridge spanning across the enclosures (Fig. S7d).

Three electrical circuits were installed to enable individual dimming of the outer and inner ring as well as the central emitter. For each enclosure, a DC power supply (MW LPF-90-24, Meanwell, Taiwan) and a three-channel LED driver were used for dimming and individual control (Vario Contro Linear Drive 180, LEDlinear GmbH, Neukirchen-Vluyn, Germany). Dimming was possible to about 0.01% of the maximum output (Fig. S6). We used an oscilloscope to test for flicker while dimmed, and the modulation frequency was found to be well above 400 Hz even for the extremely low dimming levels. A modular waterproof cable system designed for gardening applications was used (Easy Connect SAS, Toulouse, France) and modified with waterproof shrinking hose.

## Discussion

The illumination system presented here was designed to mimic skyglow at ecosystem-scale within a freshwater enclosure facility. Only a few outdoor and indoor experiments on skyglow have been performed so far. Moore et al.^30^ have investigated diel vertical migration of Daphnia but did not use an active illumination but rather enclosures that could block or expose the water in the enclosures to the surrounding urban skyglow. This is a seminal experiment, but it lacks a precise characterization of the light field and control over the treatment parameters. Very recently, Torres et al.^29^ have investigated the disturbance of a skyglow-like treatment on the migration of Talitrus saltator (sandhoppers) that normally use celestial light at night for orientation. They used a confined area at a beach and illuminated it with a single central light source with a warm white spectrum LEDs. They performed light measurements to validate their target of 0.1 lx horizontal illuminance, but the spatial variation of the light field is not provided in their letter. Kupprat et al.^28^ used a flexible light system to mimic skyglow-like illumination during the night and daylight in an indoor experiment using medium sized aquaria to test impact of ALAN on melatonin suppression. While the light source is well characterized in terms of spectral and temporal parameters, the spatial light field was not particularly taken care of, which might become important in behavioral and larger scale experiments.

In the work presented here, the aim was to produce a spatially diffuse and homogenous light field as close as possible to real world skyglow. These requirements were largely met by the system relying on LED strips emitting warm-white light with a CCT of 2700 K and a near Lambertian spatial emission pattern.

The size and architecture of the enclosure facility called for a custom solution for the lighting design, while respecting various constraints. A transdisciplinary team of scientists elaborated a solution with valuable input from lighting designers^38^. Optical modeling with raytracing enabled us to produce a tailored solution and to test light sources before purchasing materials and building prototypes or engaging in elaborate measurements. Moreover, a theoretically indefinite number of scenarios differing in geometries and other conditions can be modeled, particularly because modeling makes it possible to simulate optical properties of natural lake water (e.g. regarding scattering and absorption). In principle, the model can thus also accommodate multiple wavelengths with varying scattering and absorption properties. However, since our main objective was to optimize the spatial distribution of the light field, and since the light attenuation in the enclosure water could be effectively measured, it was sufficient to run the models for a single wavelength. Thus, a small number of basic assumptions were sufficient to simulate different geometrical options and devise an optimum solution for our illumination system in terms of optical homogeneity, minimal shading to avoid shielding of natural light, accessibility to the enclosures for sampling, practicality of implementation, and finally costs.

The final design consisted of 37 LED strips each of a length of 27.5 cm that were equally distributed along two supportive ring structures, with one LED strip being mounted at the center of the enclosure. The modeled results indicated only minimally different performance to a full-ring emitter solution (Fig. S4) and an acceptable deviation from the ideal illumination system covering the whole enclosure (see Fig. S2a,d,g). Validation of the modeling results by in situ light measurements above and below the water surface revealed only a slight mismatch between the predicted homogeneity and the measured light field distribution. The desired homogeneity of 1:2 was met at 1 m water depth, and the horizontal homogeneity was excellent at 2 m water depth. However, the light field at the water surface was slightly heterogeneous with a 1:6 intensity modulation. Therefore, any light-induced movements of organisms over small distances at the very top water layer could potentially introduce a bias in experimental results. Still, the model provided exact knowledge about this small weakness and provided potential solution. Finer spacing of the emitters in the radial direction by using a larger number of rings would have resulted in a more homogenous light field at the surface (see Fig. 2) but would compromise our design conditions. Specifically, it would have required more material and raised the building costs and time investment, as well as, most importantly, increase shading of natural light and decrease access to the enclosures for sampling. Thus, we considered our selected final design as the best compromise for the skyglow treatment.

In conclusion, this is the first time that an illumination system to mimic skyglow at ecosystem level has been achieved. Furthermore, precise optical modeling of the spatial light field distribution with physically correct light propagation was utilized for the first time during a design phase of a large-scale experiment. We succeeded in providing a highly homogeneous light field for more than 90% of the experimental volume investigated. While the illumination system itself is highly customized to the freshwater enclosure facility, the method of optical modeling and customization can be adapted to other skyglow experiments both in the laboratory and in the field. This method is particularly useful to aid experimental design for the urgently needed research question on how skyglow effects the environment, by providing exact knowledge of the full light field and by assisting the creation of tailored light distributions.

## Materials and methods

### Study site and facility

The research infrastructure to be illuminated, the LakeLab (Fig. 1b), is a large-scale experimental enclosure facility installed in Lake Stechlin, Germany. It consists of 24 enclosures, each 9 m in diameter, that are isolated from the open lake by impervious tube-shaped tarpaulin reaching down into the sediment at about 20 m depth^39^. The LakeLab is located 75 km north of the city of Berlin in one of the darkest areas of the country^18^. At this location, clear skies have near-natural brightness, and overcast nights are generally darker than clear nights^40,41^. The location is therefore excellent for testing for effects of skyglow, as the experimental controls are exposed to a near-natural night sky.

### Experimental requirements and limitations

An ideal light source to mimic skyglow would be a large hemisphere, like a planetarium dome^42^, that emits (or reflects) spatially diffuse radiation. However, such a system is unsuitable for a large outdoor facility, because it blocks natural light during the day and hampers free gas exchange between the water surface and the atmosphere. Furthermore, the spectrum of skyglow is composed of the spectra of multiple light sources in the first place. Furthermore, these light spectra are altered by being reflected at obstacles (the ground, buildings, vegetation etc.) and subsequently by wavelength dependent scattering within the atmosphere. The spectrum of skyglow will therefore be different at different places on Earth and vary over time, which is too difficult to resemble at an outdoor facility of the scale of LakeLab. Both approaches could however be implemented in future indoor studies. Therefore, acknowledging unavoidable trade-offs, we defined the following experimental requirements:

- A highly diffuse light field with incident light received from nearly all angles
- An intensity modulation better than 2:1 at 1 m depth and nearly homogeneous illumination at 2 m depth
- Light emitters with a nearly uniform (Lambertian) spatial emission pattern
- A light spectrum resembling modern outdoor lighting
- An adjustable illuminance, dimmable within the range of 0.01–10 lx at the water surface
- Minimum blocking of natural light sources such as sunlight, moonlight and starlight
- An open water surface to ensure free gas exchange with the atmosphere
- Easy access to the enclosures to sample water, deploy measurement devices, etc.

Additional practical criteria were suitability for outdoor use, including waterproofness of the light emitters, low power consumption, long lifetime, and low maintenance, electrical and chemical hazard avoidance, and total costs. The rationale behind the illuminance range was that a natural clear night has an illuminance of ca. 0.001 lx, significant skyglow starts at ca. 0.01 lx and extreme skyglow for urban cloudy skies can be more than 1 lx^20^. The target of 10 lx was set because skyglow is increasing exponentially and to have a bit of headroom with the illumination system. Please note that that despite lux is an SI unit it is non-ideal for ecological studies, particularly if no spectrum of the light source is provided. For a discussion on this see a recent paper on long-term studies of insects and ALAN^44^.

### LED strips

We opted for LEDs because they are energy-efficient, can be operated with low Voltage direct current, have excellent dimming properties, and are now widely used for outdoor lighting. LEDs are also available with many spectra and correlated color temperatures (CCTs)^43^. We selected waterproof LED strips (VarioLED Flex NIKE LD4 827 SV, LEDlinear GmbH, Neukirchen-Vluyn, Germany) with a warm-white emission spectrum and a CCT of 2700 K (Fig. S8a), which is close to typical CCTs reported for urban light polluted skies in and near Berlin^20^. The manufacturer embedded the LEDs in a scattering matrix, to produce a spatially diffuse light field with a nearly Lambertian (constant apparent brightness independent of observer’s position) spatial emission pattern (Fig. S8b). The LED strips were available in lengths between 27.5 cm and 5 m, and had a nominal luminous flux per length of 130 lm/m. In the final design, the shortest length of 27.5 cm was used.

### Raytracing model

The underwater light field was modeled using the commercial raytracing software TracePro (Version 7.7.1. Lambda Research, Littleton, CO, USA https://lambdares.com/tracepro/), which includes physically correct functions for refraction, scattering and absorption. TracePro utilizes non-sequential raytracing based on a Monte-Carlo technique. The software is widely applied to model the propagation of light in tissue, but also used for the design of complex optics or illumination^45^. Monte-Carlo raytracing works by simulating the paths of individual photons (or groups of photons) from a source to some ending position^46^. First, an individual photon is assigned a position in space (x,y,z) corresponding to a source. These starting locations are weighted according to the size and the brightness of the sources, and a position is then randomly chosen according to these weights. Next, the photon is assigned a propagation direction based on the spatial emission properties of the light source. The photon is then taken forward in a set of steps in this direction, until it is either absorbed, experiences a scattering event, or hits a boundary (e.g. a wall, the water surface, or the end of the simulation boundary). At a boundary, the photon’s direction is changed, based on the physics of the interaction. Imaginary boundaries may also be inserted into the code to determine the flux of photons at a given location. Photons in the water are scattered via a variety of physical processes, mainly Rayleigh-like scattering from molecules (sometimes also referred to as Einstein–Smoluchowski scattering), and Mie-like scattering from larger water constituents. In a Mie scattering event that dominates in lake water, the photon is diverted to a new trajectory according to a scattering phase function, which is the Henyey-Greenstein function in TracePro:

$$p\left( {\cos\uptheta } \right) = \frac{1}{2}\frac{{1 - g^{2} }}{{\left( {1 + g^{2} - 2g \cdot \cos\uptheta } \right)^{3/2} }},$$

where $p\left( {\cos\uptheta } \right)$ is the probability density function of the cosine of θ, with θ being the angle relative to the original propagation direction and g parameterizes the anisotropy of scattering, taking values between + 1 and − 1 with positive values indicating forward and negative values backward scattering.

TracePro is based on computer aided design (CAD) modeling, which involves first creating the geometrical environment composed of discrete elements, and then applying the appropriate material properties to each of these elements. To model illumination in an enclosure, we thus created a water-filled cylinder of the size of the enclosures (Fig. S9a) adding ring shaped light emitters above the water surface, walls and bottom layers with a specific reflectivity and various detector planes (Fig. S9b). The rays were then propagated through the water in the cylinder (Fig. S9c). The spatial emission pattern of the LED strips (using the photometric data files from the manufacturer, see Fig. S8b) was applied to the lower part of the lighting ring facing the water. Material constants such as the refractive index of water (n_water_ = 1.33) and the absorption coefficient $\mu_{a}$ and scattering coefficient $\mu_{s}$ were applied to the water-filled cylinder. Although the absorption of pure water is a built-in feature of the software, the optical properties from optically active constituents (phytoplankton, colored dissolved organic matter etc.) of freshwater bodies need to be applied. The modeling parameters were set as follows: the anisotropy factor was g = 0.9 to simulate forward scattering, the scattering coefficient was $\upmu _{{\text{s}}} = 1/{\text{m}}$ and the absorption coefficient was ${\upmu }_{{\text{a}}} = 0.55/{\text{m}}$ to represent a wavelength of 500 nm. These values are on the order of previously measured values for the lake^47^, while our own measurements for the diffuse downwelling attenuation coefficient ${\text{k}}_{{\text{d}}}$ ranged between 0.3/m and 1.5/m during summer months. Since the focus was on the spatial distribution of light in the enclosures, the modeling was performed for monochromatic light only.

The is plausible because the scattering will not vary as dramatically with wavelength as the absorption in natural freshwaters like Lake Stechlin. In pure water the light scattering coefficient is higher for short wavelengths than for longer wavelengths, similar to Rayleigh scattering within the atmosphere. However, it is in the range of $\upmu _{{{\text{s}},{\text{pure}}}} = 0.01/{\text{m}}$ for wavelengths above 400 nm^48^. The scattering coefficient from the optical constituents in natural freshwaters in form of organic and inorganic particles will be much higher (we used $\upmu _{{\text{s}}} = 1/{\text{m}}$ at 500 nm, which is representative for Lake Stechlin) but will not vary much with wavelength for such water bodies^49^. We tested this for two other wavelengths 400 nm ($\upmu _{{\text{s}}} = 1.3/{\text{m}}$, $\upmu _{{\text{a}}} = 0.9/{\text{m}}$) and 600 nm ($\upmu _{{\text{s}}} = 0.8/{\text{m}}$, $\upmu _{{\text{a}}} = 0.7/{\text{m}}$) as well as a scenario with strong absorption and relatively low scattering ($\upmu _{{\text{s}}} = 0.8/{\text{m}}$, $\upmu _{{\text{a}}} = 3/{\text{m}}$). The results are shown in Fig S10 for 1 m and 2 m depth. Hardly any differences can be perceived for the different wavelengths but a slight deviation at 1 m depth for the strong absorption can be seen. Thus, for a freshwater system like Lake Stechlin, the single wavelength approach is acceptable. However, this can change in strongly absorbing waters and in very turbid waters, where the scattering coefficient can have a stronger wavelength dependency^49^.

## Data availability

All data are available in the main text or the supplementary materials.

## Supplementary Information

Supplementary Information.
