# Supplementary material for: Design and implementation of an illumination system to mimic skyglow at ecosystem level in a large-scale lake enclosure facility
Source: Sci Rep. 2021 Dec 6;11:23478. doi: 10.1038/s41598-021-02772-4 (PMC8648721; doi:10.1038/s41598-021-02772-4)
Supplement: Supplementary file 1 — Supplementary Information. [file 41598_2021_2772_MOESM1_ESM.docx]

**Supplement to: Design and implementation of an illumination system to mimic skyglow at ecosystem level in a large-scale lake enclosure facility**

The light propagation model allowed to test different geometries than the pre-considered circular geometry (Fig. S5). In a plausibility test, also a rectangular geometry was considered because it probably is easier to manufacture rectangular than circular structures. Furthermore, the ideal light field in form of a homogeneous diffuse light field was modeled (Fig. S6 a,d,g) as a benchmark for the circular light sources. The rectangular architecture was not further considered because of the poor results at the edges of the enclosures.


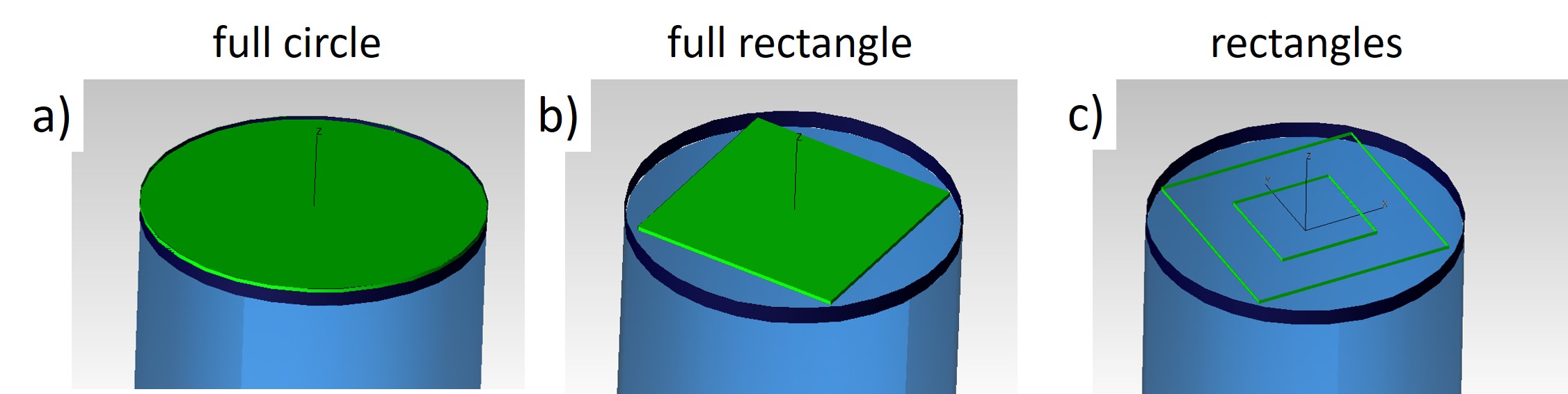


**Figure S1 CAD models for the single circular emitter spanning over the full enclosure (a), a full rectangular emitter (b) and two rectangular emitters (c).**


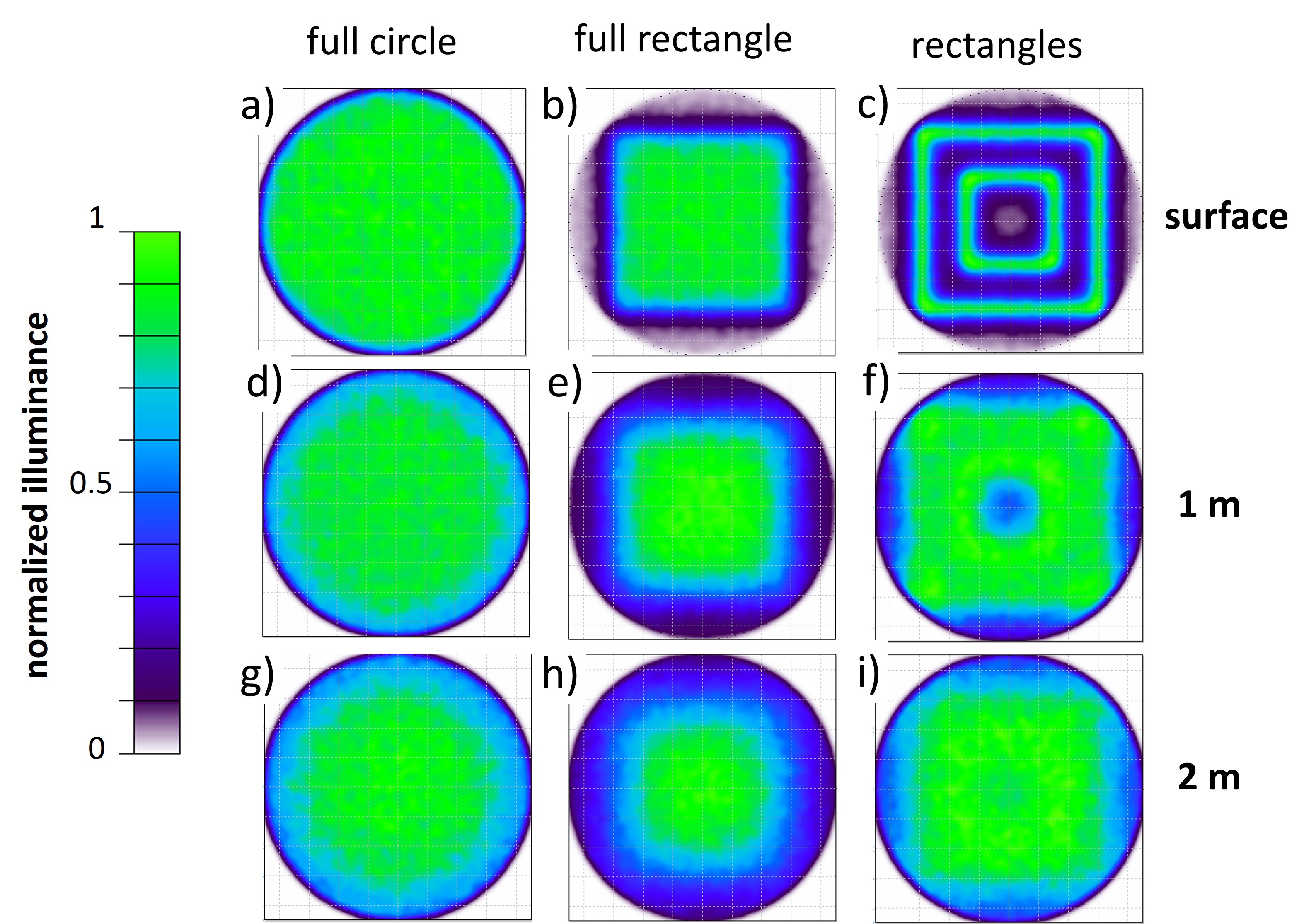


**Figure S2 Modelled illuminance distribution for additional geometries: an ideal homogeneously distributed light field above the water surface from a single circular emitter spanning over the full enclosure (a,d,g), a full rectangular emitter (b,e,h) and two rectangles (c,f,i).**


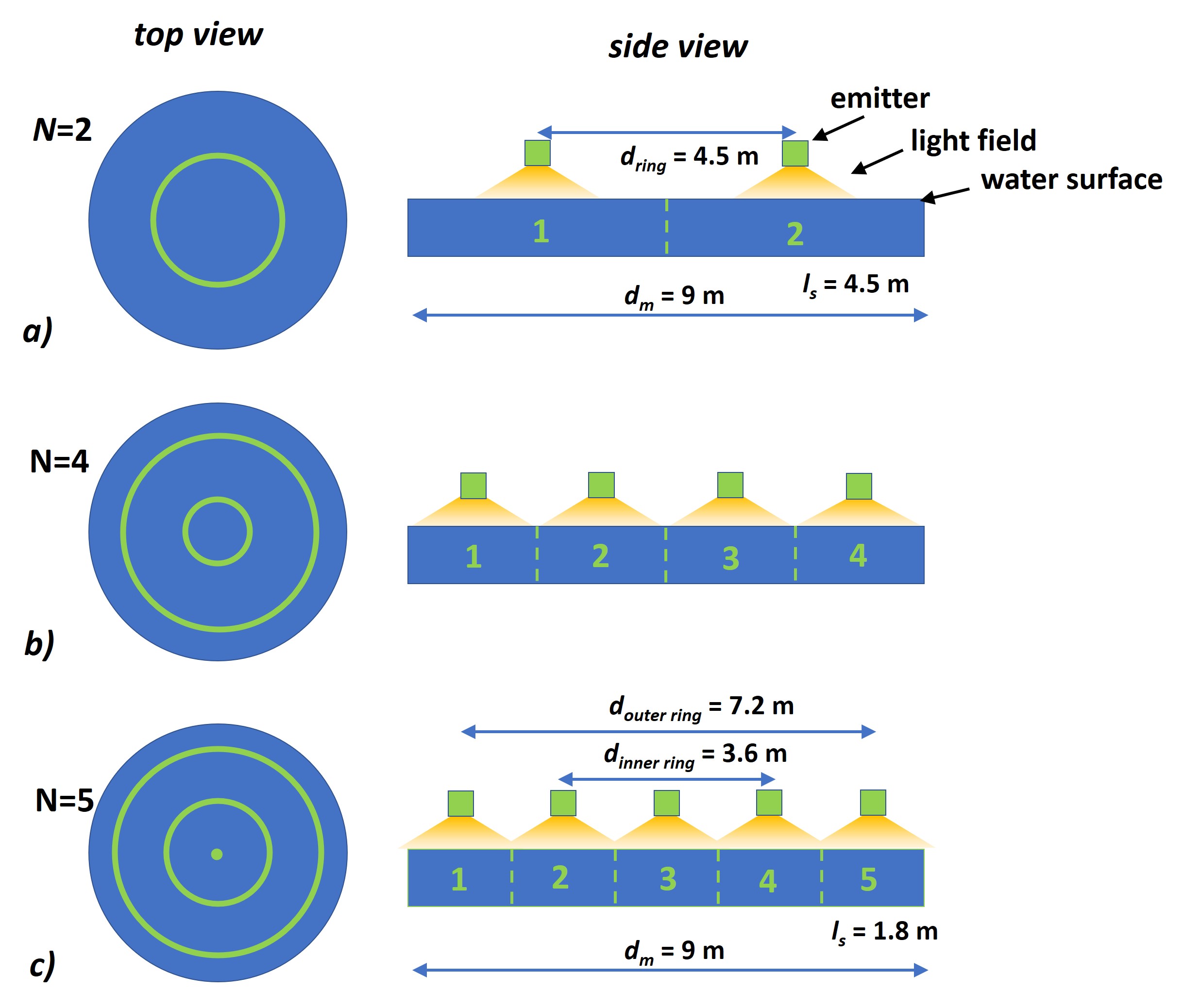


**Figure S3: Sectioning of the enclosure along the diameter into N segments for different ring geometries.**


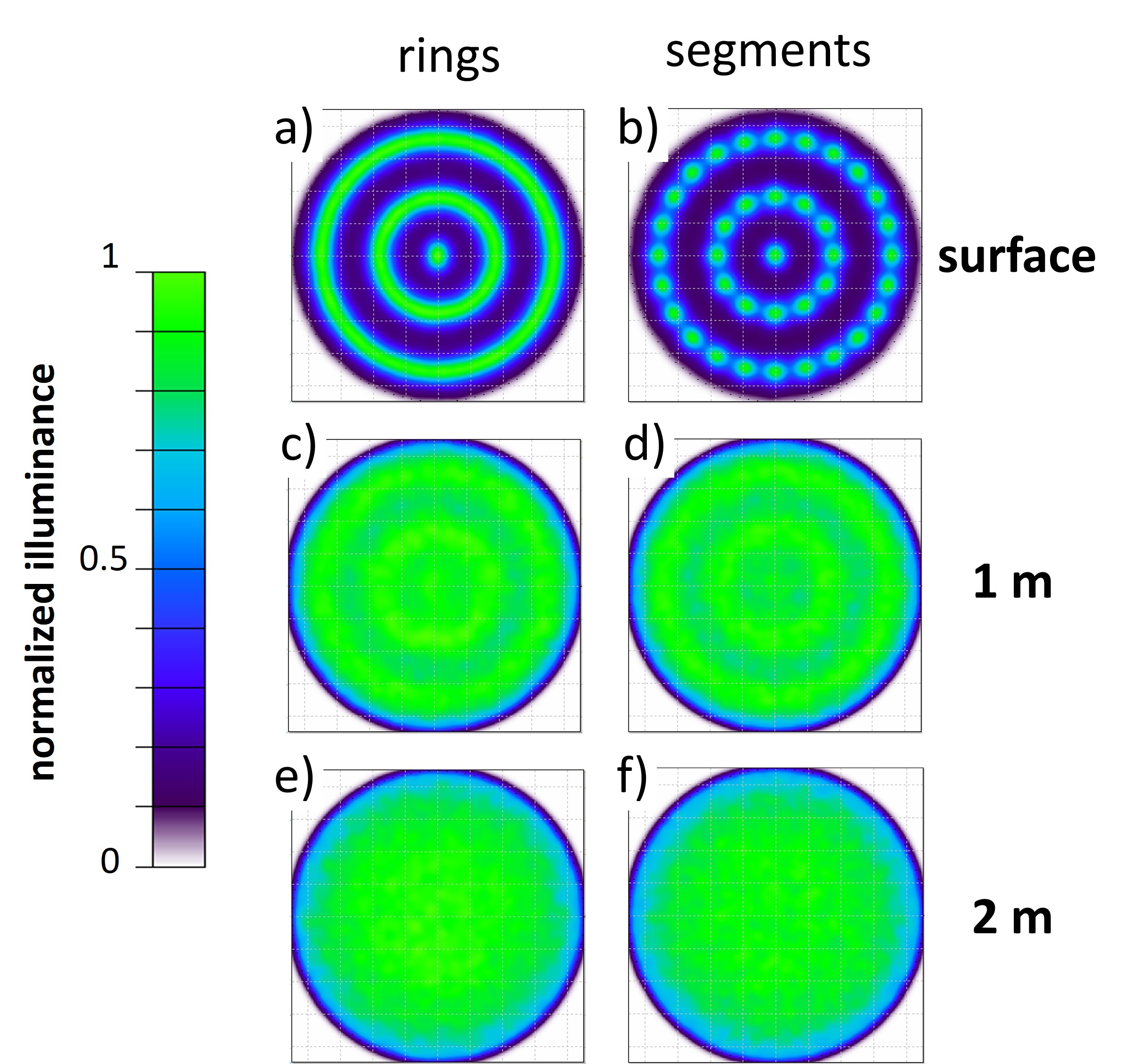


**Figure S4: Simulation comparing the full ring geometry (a,c,e) with the geometry sectioned along the azimuth using 37 individual LED strips (b,d,f). The horizontal light distribution is shown in the upper row (a,b) for the water surface, in the middle row (c,d) for 1 m and in the lower row (e,f) for 2 m depth. Apart from the surface, the results are equivalent.**

**
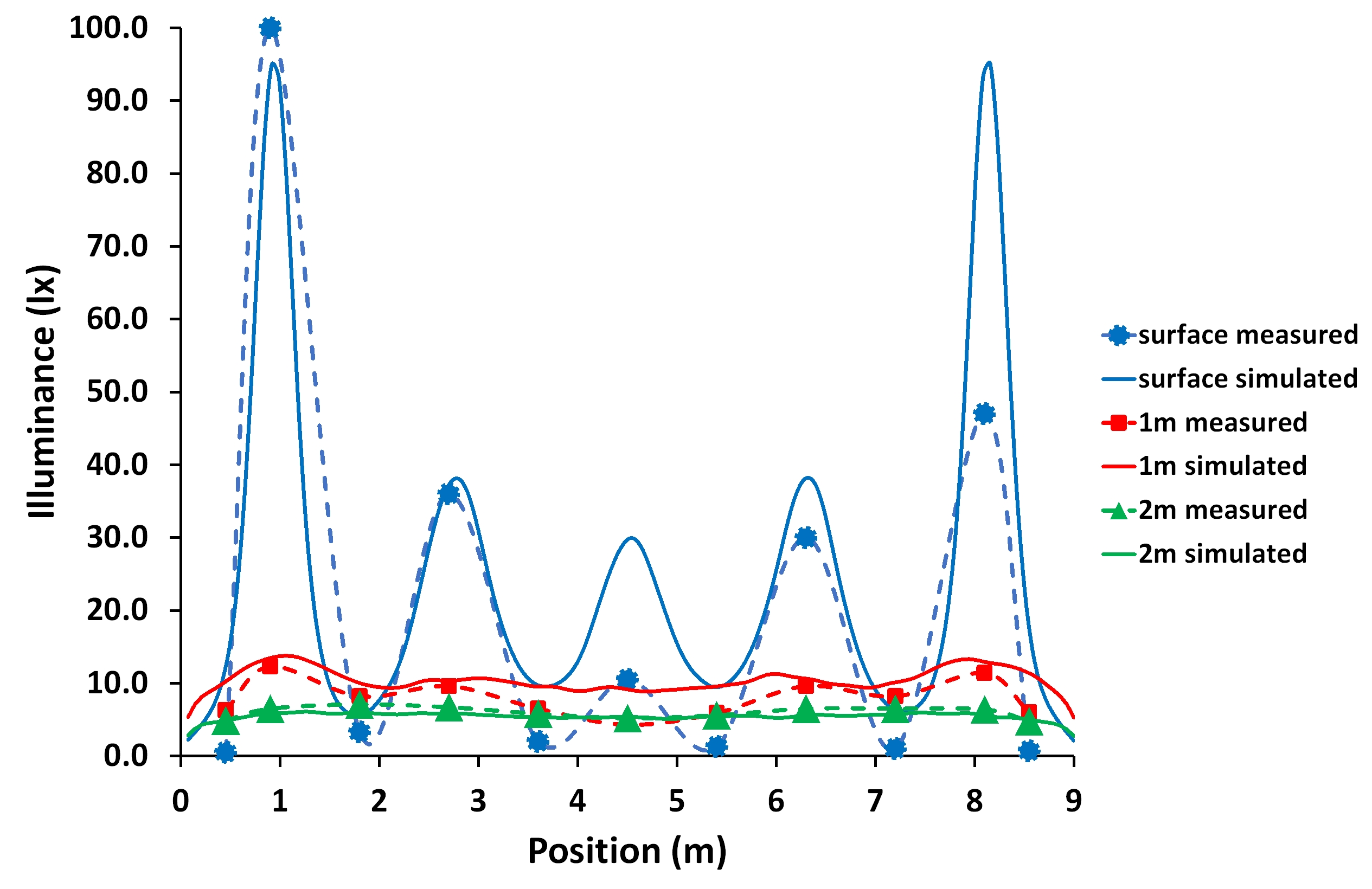
**

**Figure S5: Measured (dashed lines) and modeled (solid lines) illuminance distribution at the surface (blue circles), 1 m (red aqures) and 2 m (green triangles) water depth for the unoptimized setup at full output power without the additional white wall installed.**

**Table S1 Measured horizontal illuminance (in lx) at the surface, 1 m and 2 m for the non-optimized setup operating at full output flux.**

|  | Measurement point | | | | | | | | | | | | | | |  | |  | |  |
| --- | --- | --- | --- | --- | --- | --- | --- | --- | --- | --- | --- | --- | --- | --- | --- | --- | --- | --- | --- | --- |
|  | 1 | 2 | 3 | | 4 | 5 | | 6 | 7 | 8 | 9 | 10 | | 11 | | avg | | SD | | max/min |
| depth |  | | |  | | | illuminance | | | (lx) | | |  | |  | |  | |  |  |
| surface | 0.5 | 99.9 | 3.3 | | 36.0 | 2.0 | | 10.5 | 1.3 | 30.0 | 1.0 | 47.0 | | 0.7 | | 21.1 | | 29.6 | | 200 |
| 1m | 6.3 | 12.3 | 8.2 | | 9.6 | 6.5 | | 4.3 | 5.9 | 9.6 | 8.2 | 11.4 | | 6.0 | | 8.0 | | 2.4 | | 2.9 |
| 2m | 4.9 | 6.5 | 7.1 | | 6.7 | 5.8 | | 5.2 | 5.6 | 6.5 | 6.5 | 6.4 | | 4.8 | | 6.0 | | 0.7 | | 1.5 |

**Table S2 Measured horizontal illuminance (in lx) measured at the surface, 1 m and 2 m for the optimized setup with some LEDs dimmed operating near full output flux.**

|  | Measurement point | | | | | | | | | | | | | | |  | |  | |  |
| --- | --- | --- | --- | --- | --- | --- | --- | --- | --- | --- | --- | --- | --- | --- | --- | --- | --- | --- | --- | --- |
|  | 1 | 2 | 3 | | 4 | 5 | | 6 | 7 | 8 | 9 | 10 | | 11 | | avg | | SD | | max/min |
| depth |  | | |  | | | illuminance | | | (lx) | | |  | |  | |  | |  |  |
| surface | 9.2 | 35.1 | 1.7 | | 25.6 | 7.3 | | 13.8 | 3.6 | 28.4 | 9.3 | 41.5 | | 9.9 | | 16.9 | | 12.9 | | 24.4 |
| 1m | 9.0 | 11.0 | 6.0 | | 8.7 | 8.6 | | 5.6 | 6.0 | 8.9 | 7.7 | 10.3 | | 8.7 | | 8.2 | | 1.7 | | 2.0 |
| 2m | 5.1 | 5.7 | 6.2 | | 5.9 | 5.4 | | 5.2 | 5.3 | 5.9 | 5.9 | 5.9 | | 5.2 | | 5.6 | | 0.4 | | 1.2 |

**Table S3 Measured horizontal illuminance (in lx) measured at the surface, 1 m and 2 m depth for the optimized setup dimmed to the high skyglow treatment using individual dimming levels for the two rings and the central emitter.**

|  | Measurement point | | | | | | | | | | | | | | |  | |  | |  |
| --- | --- | --- | --- | --- | --- | --- | --- | --- | --- | --- | --- | --- | --- | --- | --- | --- | --- | --- | --- | --- |
|  | 1 | 2 | 3 | | 4 | 5 | | 6 | 7 | 8 | 9 | 10 | | 11 | | avg | | SD | | max/min |
| depth |  | | |  | | | illuminance | | | (lx) | | |  | |  | |  | |  |  |
| surface | 2.6 | 12.2 | 2.1 | | 9.3 | 2.8 | | 10.8 | 2.5 | 8.9 | 2.6 | 11.8 | | 2.8 | | 6.2 | | 4.1 | | 5.8 |
| 1m | 2.5 | 3.1 | 2.5 | | 2.4 | 1.9 | | 2.2 | 1.8 | 2.4 | 2.1 | 2.8 | | 2.4 | | 2.4 | | 0.4 | | 1.7 |
| 2m | 1.6 | 1.7 | 1.7 | | 1.8 | 1.6 | | 1.7 | 1.7 | 1.7 | 1.7 | 1.8 | | 1.7 | | 1.7 | | 0.1 | | 1.1 |

**Figure S6: Dimming curve for the LED strips.**

**
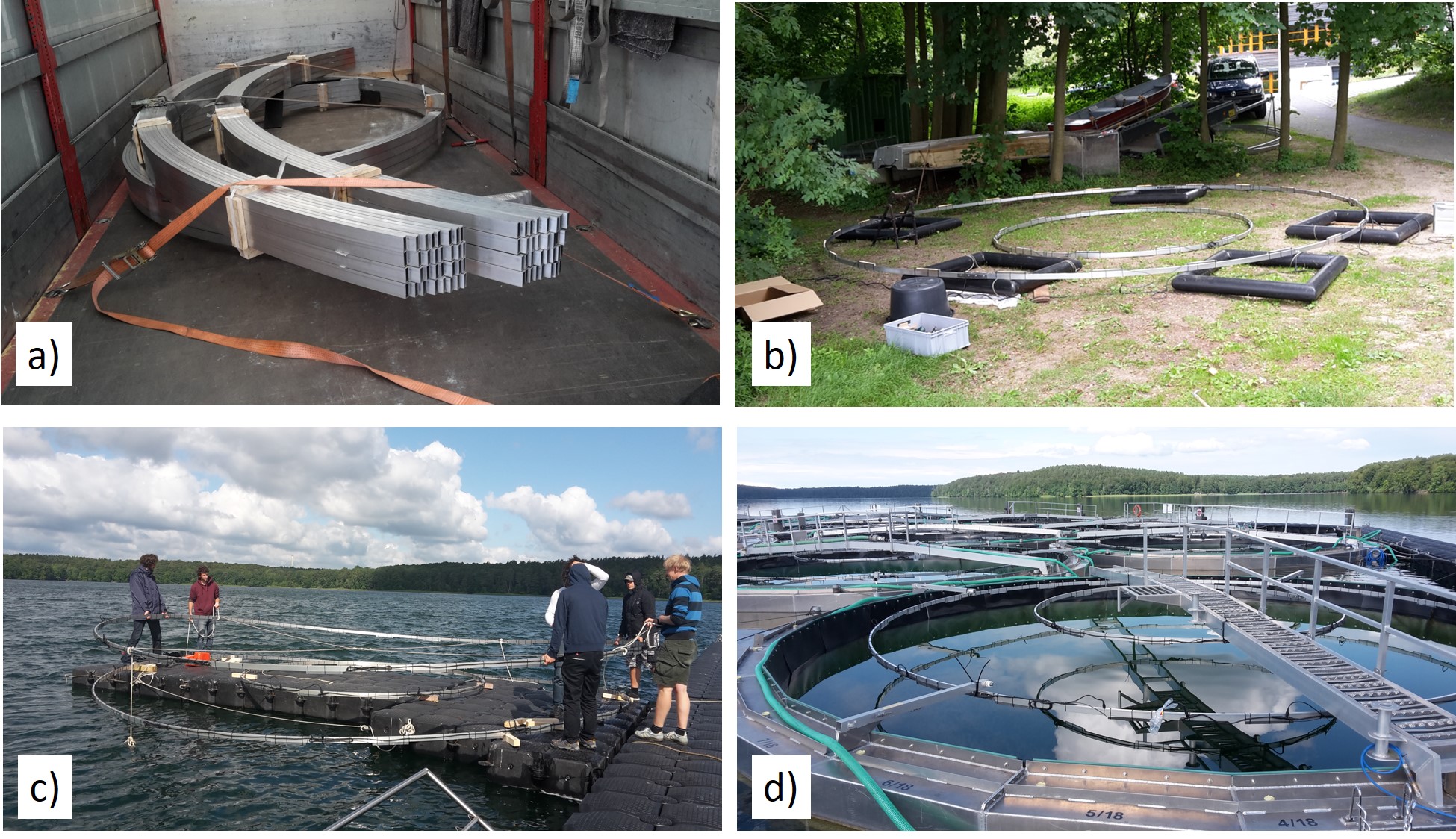
**

**Figure S7 The mechanical support structure a) rectangular aluminum tubes, bent and delivered in segments b) assembly on land, and c) final construction mounted on an enclosure.**


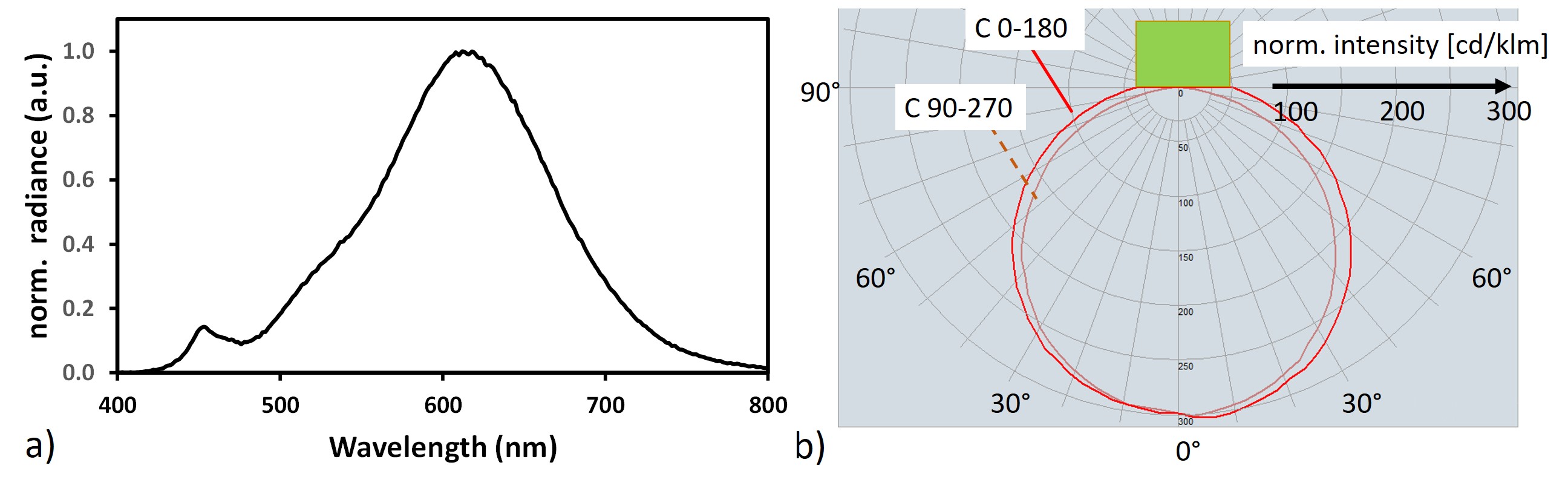


**Figure S8: Emission characteristics of the LED strip, VarioLED Flex NIKE LD4 827, a) spectral radiance of the LED emission (normalized to the peak wavelength) with a CCT of 2700 K and b) spatial emission pattern of the LED light in a photometric polar diagram using information provided by the manufacturer. The green square represents the LED emitter.**


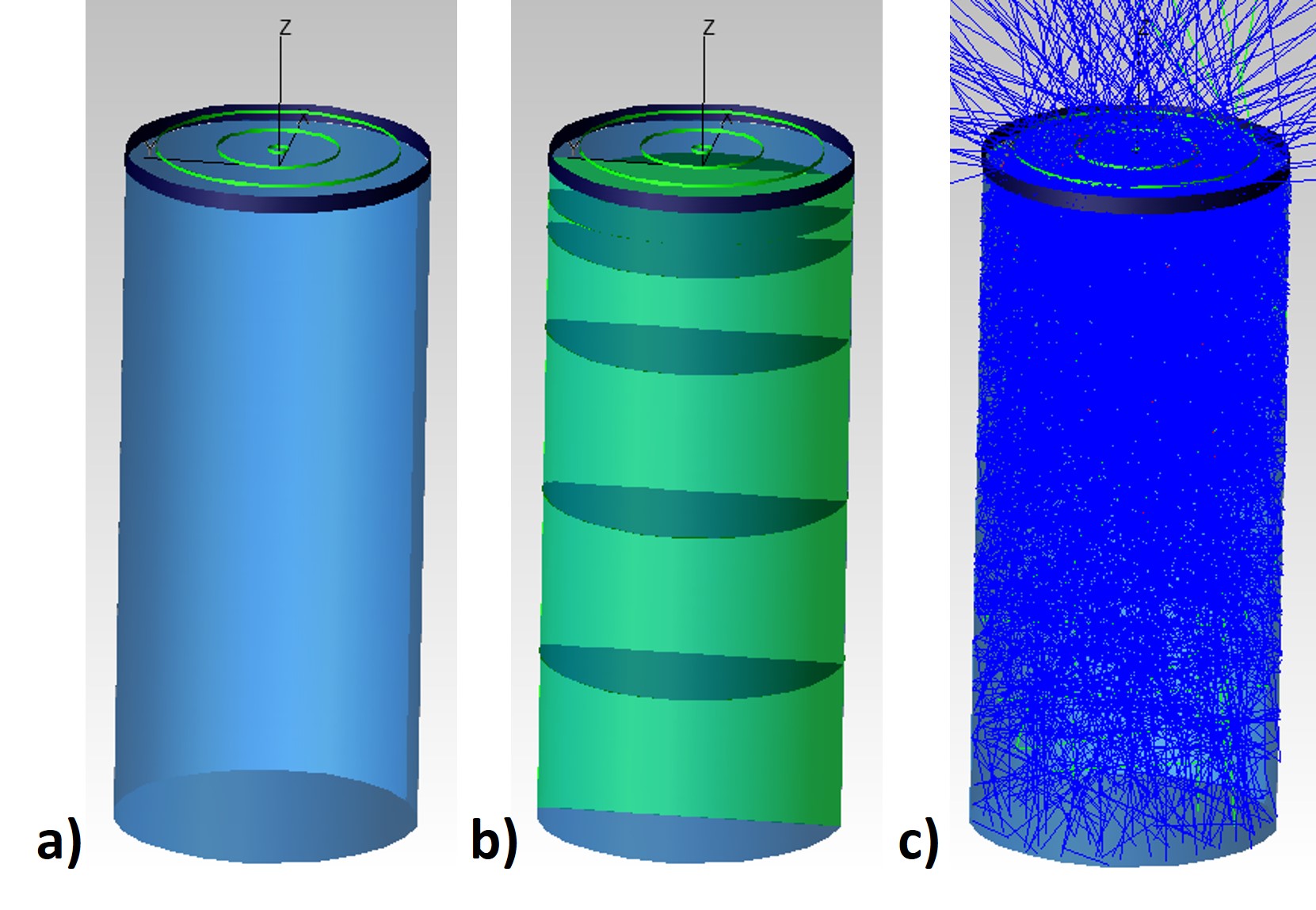


**Figure S9: a) CAD model of a water-filled enclosure 9 m in diameter and 20 m deep with ring-shaped light emitters above the water surface (bright green), b) with added multiple detector planes (green and grey planes), and c) additionally showing a part of the individual traced rays.**

**
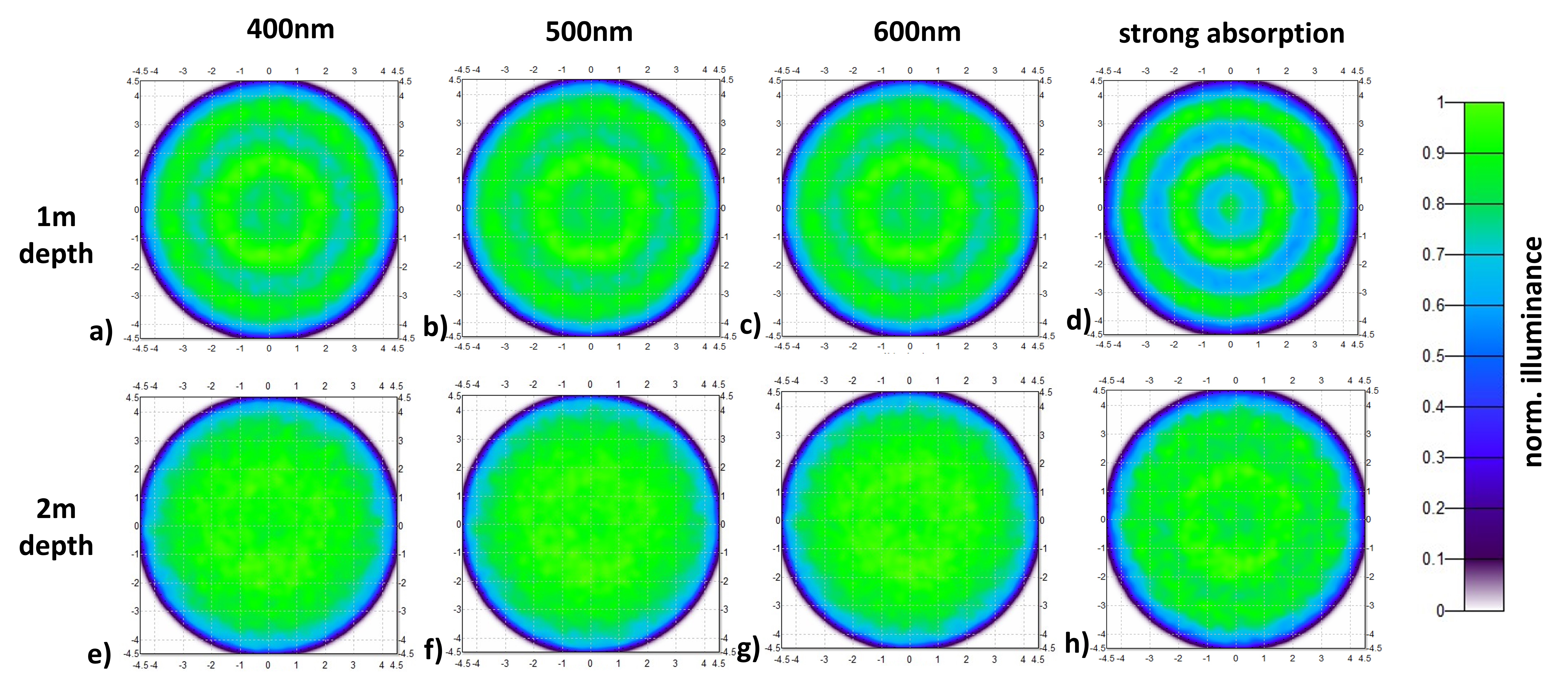
**

**Figure S10: Modeling results for different absorption and scattering parameters or wavelengths respectively.**
